# ASSOCIATIONS BETWEEN ENVIRONMENT AND COGNITIVE FUNCTIONING FOR OLDER ADULTS: A CASE STUDY OF SINGAPORE

**DOI:** 10.1093/geroni/igad104.1249

**Published:** 2023-12-21

**Authors:** Yuezhong Liu, Chek Hooi Wong, Ringo Ho, Yin-Leng Theng

**Affiliations:** Nanyang Technological University, Singapore, Singapore; Research for Impact, Singapore, Singapore; Nanyang Technological University, Singapore, Singapore; Nanyang Technological University, Singapore, Singapore

## Abstract

This study aims to examine the interplay between the intrinsic capacity of older adults and built environmental characteristics, and how these factors influence their outside daily life gait speed (DGS) in neighbourhoods. We collected and analyzed 33 participants who travelled a total of 2,428 kilometres and took a combined 1,428,793 steps outside of their homes over a period of 220 days in Singapore. Mini-Mental State Examination (MMSE) cut-offs (accounting for education attainment) were used to distinguish between those with cognitive impairment (CI) and those without cognitive impairment (Non-CI). The mean (SD) age of the participants was 69.2 (7.14), and 21 (64%) of them were female. 14 (42%) participants were identified as having cognitive impairment. A high Gross Plot Ratio was found to be associated with a lower DGS for both CI (b=-0.03, t = 0.19, p < .01) and Non-CI (b=-0.03, t = 0.16, p < .01). The DGS of individuals with CI was generally slower compared to Non-CI, with the greatest difference observed in business areas where the Non-CI group walked at a speed twice as fast. Individuals with CI had a slower DGS and required more adaptation to the environment. They also experienced lower quality of life-space interaction and relied more on vehicular travel from the density and frequency of activity points. Creating an environment that requires less attention and reduces cognitive load may help promote safe walking among individuals with CI and compensate for their reduced ability to perceive environmental risk.

